# Treatment patterns and low-density lipoprotein cholesterol (LDL-C) goal attainment among patients receiving high- or moderate-intensity statins

**DOI:** 10.1007/s00392-017-1193-z

**Published:** 2017-12-22

**Authors:** Kathleen M. Fox, Ming-Hui Tai, Karel Kostev, Maximilian Hatz, Yi Qian, Ulrich Laufs

**Affiliations:** 1 Strategic Healthcare Solutions LLC, Aiken, SC USA; 2 Amgen Inc, Thousand Oaks, CA USA; 3 QuintilesIMS, Frankfurt, Germany; 4 Klinik und Poliklinik für Kardiologie, Universitätsklinikum Leipzig, Leipzig, Germany

**Keywords:** Cardiovascular disease, LDL-C, Hyperlipidemia, Statin, LDL-C goal attainment

## Abstract

**Background:**

European clinical guidelines recommend a low-density lipoprotein cholesterol (LDL-C) goal of < 70 mg/dL. Statin use varies and past studies suggest low rates of real-world goal attainment. This study describes LDL-C goal attainment among atherosclerotic CV disease (ASCVD) patients with various utilization patterns of moderate- or high-intensity statins in routine care.

**Methods:**

This retrospective cohort study used electronic medical records data from the QuintilesIMS® Disease Analyzer (> 2 million individuals annually) to identify ASCVD (coronary atherosclerosis, stable/unstable angina, myocardial infarction, ischemic stroke, transient ischemic attack, aneurysm, peripheral artery disease) patients on moderate-/high-intensity statin in Germany. Proportion of patients with LDL-C < 70 mg/dL was determined using the lowest LDL-C value for each patient (index) in 2012, 2013, and 2014, while on statin. Treatment patterns were assessed for patients with at least 1 year of post-index follow-up. Results were stratified by year and treatment pattern [no change, switch, dose up-/down-titration, discontinuation (≥ 90 day gap)].

**Results:**

In > 14,000 patients assessed in each year (mean age 71 years, 35% female, 8–12% taking high-intensity statins), approximately 80% had LDL-C ≥ 70 mg/dL. Treatment patterns were assessed for most (88–93%) patients. Approximately 79–81% of patients made no change to statin regimens, 1% switched statins, 14–16% discontinued; 1% of moderate-intensity patients up-titrated, and 3% of all patients down-titrated. LDL-C goal attainment in these treatment pattern groups was 20, 16–24, 17, 11–14, and 17–19%, respectively.

**Conclusions:**

Majority of ASCVD patients had LDL-C ≥ 70 mg/dL while on moderate-/high-intensity statins. Despite low LDL-C goal attainment, few patients changed their treatment regimens.

**Electronic supplementary material:**

The online version of this article (10.1007/s00392-017-1193-z) contains supplementary material, which is available to authorized users.

## Introduction

The benefit of lowering low-density lipoprotein cholesterol (LDL-C) is well documented in patients with hyperlipidemia, with strong evidence of decreases in both all-cause mortality and the occurrence of major cardiovascular (CV) outcomes [1–5]. Much of this evidence has come from clinical trials of moderate and intensive statin therapy in patients with atherosclerotic cardiovascular disease (ASCVD) who are generally considered to be at particularly high risk of cardiovascular events, as well as in patients without prior CV history.

The European Society of Cardiology (ESC)/European Atherosclerosis Society (EAS) guidelines, for example, recommend tailoring treatment to each patient’s level of cardiovascular risk [6–8]. These guidelines have set an LDL-C goal of < 70 mg/dL for patients considered to be at very high cardiovascular risk. Patients in this risk category have documented cardiovascular disease (e.g., prior myocardial infarction [MI]), or a 10% or greater 10-year risk of fatal cardiovascular disease, and in randomized controlled trials, the guideline-specified LDL-C goal has been shown to reduce the risk of recurrent cardiovascular events [7].

In routine clinical practice, statin intolerance and other factors may lead to treatment discontinuation, switching among statin agents, dose adjustments, or the need for augmentation of the statin regimen with additional therapies. These changes are common, and have the potential to impact real-world LDL-C goal attainment and the therapeutic benefits that are achieved by statin users outside of clinical trials [9–13]. In Europe, for example, statin use varies by country, and LDL-C goal attainment is suboptimal in routine practice, even among patients with known ASCVD [14–16]. Several studies have previously evaluated LDL-C goal attainment, but insights from those studies are limited by generally small sample sizes and limited recent data [17, 18]. This study was undertaken to provide recent real-world data on achievement of LDL-C < 70 mg/dL in a large population of ASCVD patients using moderate- or high-intensity statins overall, and in patients with different statin treatment patterns (e.g., switching, dose titration, discontinuation).

## Patients and methods

### Data source and study population

This is a retrospective cohort study with data obtained from the QuintilesIMS^®^ Germany Disease Analyzer (QuintilesIMS^®^ DA) for January 1, 2012 and December 31, 2014. This database contains over 14 million anonymized electronic medical records from general practitioners and specialists who represent 2.4% of all medical practices in Germany. Patients in the database are representative of the whole German population with respect to age, geography, and treatment characteristics. The study included adults with ASCVD who used moderate- or high-intensity statins during the study period. ASCVD was defined by ICD-10 codes for aneurysm, cerebrovascular disease, coronary atherosclerosis/angina/old myocardial infarction (MI), ischemic stroke (IS), MI, peripheral artery disease (PAD), transient ischemic attack (TIA), unstable angina (UA), and atherosclerosis of the arterial bed not previously defined. The definitions of moderate-intensity (atorvastatin < 30 mg or equivalent) and high-intensity (atorvastatin ≥ 30 mg or equivalent) statin regimens used in this study were adapted from the 2013 American Heart Association (AHA) and American College of Cardiology (ACC) guidelines on the treatment of hyperlipidemia (Supplementary Table 1) and have been previously published [19].

The study cohorts were identified for each calendar year (i.e., 2012, 2013, and 2014) to examine variations in goal attainment over time in a largely stable patient population. Each cohort included patients who met the following criteria: (1) at least one LDL-C value in the given year, with the index date set to the date of the lowest LDL-C value obtained in that year, (2) a prescription for a moderate- or high-intensity statin with days supply that overlapped with the index date, and (3) medical records available for the 12 months prior to the index date (baseline period). All qualified patients were included in analysis of LDL-C goal attainment. To examine statin treatment patterns, we restricted the study cohorts to patients with medical records available for at least 12 months following the index date.

### Statistical analysis

Descriptive statistics were used to summarize patient demographics (age, sex, insurance type) and baseline clinical characteristics including Charlson Comorbidity Index scores [20], and to describe the presence/absence of key conditions of interest (i.e., diabetes, hypertension, coronary heart disease, myocardial infarction, ischemic stroke, peripheral artery disease, chronic kidney disease, chronic obstructive pulmonary disease, heart failure, and depression).

**Fig. 1:**
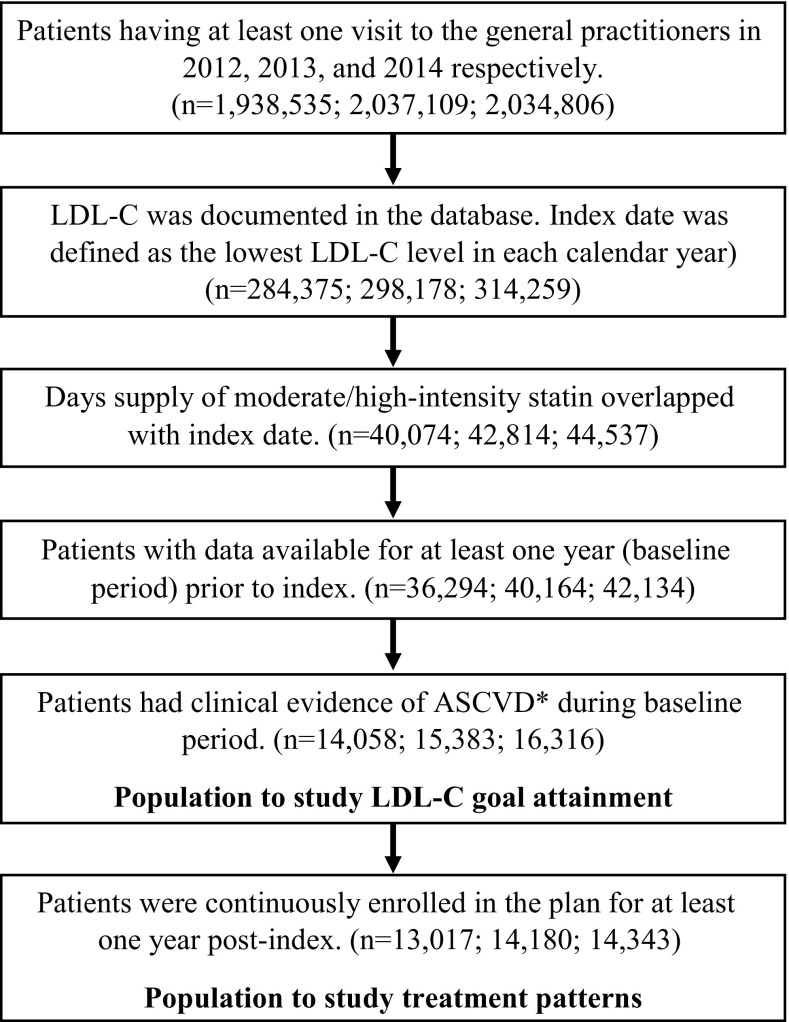
Patient selection for annual cohorts of patients with atherosclerotic cardiovascular disease (ASCVD) using moderate-/high-intensity statins

Treatment patterns [i.e., discontinuation (defined as failure to refill statin prescriptions within 90 days of end-of-day’s supply), switching between statins, up- and down-titration (defined as at least one increase or decrease of statin dosage), and time to treatment modification (defined as the time from index to the treatment pattern changes for patients)] were analyzed for the subgroup of patients who also had at least 1 year of post-index follow-up (Fig. 1); these patients were followed through the earliest of December 31, 2015 or lost to follow-up. In addition, the percentage of patients attaining the LDL-C goal of 70 mg/dL in each calendar year was determined using LDL-C data obtained on the index dates. This approach provided a reasonable and conservative assessment of goal attainment since patients were not required to remain at goal, but were counted as ‘successful’ if they reached the LDL-C goal at any point while on statin therapy in the year being assessed. Goal attainment was determined for the study population overall and for patients stratified by statin intensity and the comorbidities listed previously. For patients included in the treatment pattern analysis, goal attainment was assessed for the subgroups of patients exhibiting each treatment pattern.

## Results

The number of patients meeting study inclusion criteria was 14,058 in 2012, 15,383 in 2013, and 16,316 in 2014 (Fig. 1). In these three annual cohorts, the mean age was 71 years, 35% of patients were female, and 8–9% of patients had private health insurance (Table 1). At index, the majority (88–92%) of study patients were taking moderate-intensity statins and 8–12% were taking high-intensity statins. In each annual cohort, 7–10% of patients used ezetimibe. In the baseline period, hypertension and coronary heart disease were common, respectively, affecting approximately 75 and 70% of patients in each annual cohort. Just under one-third of patients in each annual cohort had been diagnosed with Type 2 diabetes.

**Table 1:** Baseline characteristics of ASCVD patients using moderate-/high-intensity statins, by annual cohort

Characteristics	2012 (*n* = 14,058)		2013 (*n* = 15,383)		2014 (*n* = 16,316)	
	*n*	%	*n*	%	*n*	%
Age, mean (SD)	70.6 (10.4)		70.6 (10.5)		70.8 (10.6)	
Female	4977	35.4	5,276	34.3	5629	34.5
Private health insurance coverage	1195	8.5	1,323	8.6	1371	8.4
High-intensity statin use	1130	8.0	1629	10.6	1956	12.0
Ezetimibe use	1136	9.5	1209	7.9	1107	6.8
Type 2 diabetes	4414	31.4	4646	30.2	5042	30.9
Hypertension	10,558	75.1	11,399	74.1	12,123	74.3
Coronary heart disease	9827	69.9	10,676	69.4	11,274	69.1
Myocardial infarction	3233	23.0	3615	23.5	3671	22.5
Ischemic stroke	731	5.2	831	5.4	946	5.8
Peripheral artery disease	2095	14.9	2184	14.2	2496	15.3
Chronic kidney disease	548	3.9	646	4.2	767	4.7
COPD	1237	8.8	1431	9.3	1566	9.6
Heart failure	1870	13.3	2046	13.3	2350	14.4
Depression	1434	10.2	1615	10.5	1811	11.1
Charlson Comorbidity Index mean (SD)	1.8 (1.4)		1.8 (1.4)		1.9 (1.5)	

*COPD* chronic obstructive pulmonary disease, *SD* standard deviation

**Fig. 2:**
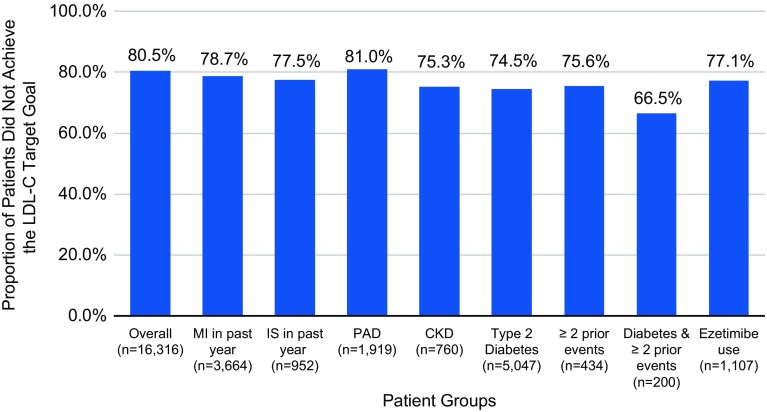
Proportion (%) of ASCVD patients using moderate-/high-intensity statins with LDL-C ≥ 70 mg/dL, full study population and subgroups by baseline clinical characteristics, 2014

In each year, approximately 80% of all study patients failed to attain LDL-C < 70 mg/dL while on moderate- or high-intensity statin therapy (Fig. 2, Supplementary Table 2). In 2014, the proportion of patients who did not achieve the LDL-C goal ranged from 66.5% in patients with diabetes and two prior cardiovascular events to 81% among patients with PAD with similar results in 2012 and 2013.

Treatment patterns were assessed for approximately 13,000 to 14,000 patients in each of the annual cohorts. In these populations, the majority (79–81%) of moderate- or high-intensity statin users remained on the same statin regimen throughout the 1 year of follow-up (Table 2). Among those on high-intensity statins, 64–75% remained on stable therapy. In the full population, 14–16% of patients discontinued high- or moderate-intensity statin therapy during follow-up, with a higher discontinuation rate (16–24%) among patients on high-intensity therapy. Only 1% of patients on moderate-intensity statins up-titrated the statin dose. Approximately 3% of all patients down-titrated their statin dose, including 9–10% of patients using high-intensity statins.

**Table 2:** Statin treatment patterns among ASCVD patients, by annual cohort

Treatment patterns	2012		2013		2014	
	*n*	%	*n*	%	*n*	%
High-intensity statin						
Total no. of patients	1045		1516		1722	
Same statin prescription and dose post-index	669	64.0	1045	68.9	1286	74.7
Other statin prescription but same dose post-index	24	2.3	18	1.2	15	0.9
Down-titrating	97	9.3	147	9.7	154	8.9
Discontinuing	255	24.4	306	20.2	267	15.5
High- or moderate-intensity statin						
Total no. of patients	13,017		14,180		14,343	
Same statin prescription and dose post-index	10,231	78.6	11,360	80.1	11,639	81.1
Other statin prescription but same dose post-index	187	1.4	189	1.3	159	1.1
Up-titrating	130	1.0	128	0.9	124	0.9
Down-titrating	385	3.0	403	2.8	374	2.6
Discontinuing	2084	16.0	2100	14.8	2047	14.3

*ASCVD* atherosclerotic cardiovascular disease

Baseline characteristics of patients exhibiting each treatment pattern are presented in Table 3. Among patients who discontinued moderate-/high-intensity statin use, the mean age was 71 years, 74% had hypertension, 16% had PAD, and 14% had heart failure, which is similar to baseline characteristics of patients who remained on the same statin. Patients who increased their statin dose (up-titration, *n* = 124) were younger on average than patients who remained on the same statin, and the up-titrated group had a lower proportion of patients with diabetes compared with patients who exhibited other treatment patterns.

**Table 3:** Statin treatment patterns and baseline characteristics of ASCVD patients using moderate-/high-intensity statins, 2014

Baseline characteristics	Treatment pattern									
	Same statin (*n* = 11,639)		Switched statin (*n* = 159)		Down-titration (*n* = 374)		Up-titration (*n* = 124)		Discontinuation (*n* = 2,047)	
	*n*	%	*n*	%	*n*	%	*n*	%	*n*	%
Age in years, mean (SD)	70.8 (10.4)		68.4 (10.8)		66.8 (10.4)		66.0 (10.4)		71.0 (10.4)	
Female	4027	34.6	60	37.7	103	27.5	34	27.4	731	35.7
Type 2 diabetes	3655	31.4	47	29.6	91	24.3	21	16.9	612	29.9
Hypertension	8706	74.8	112	70.4	288	77.0	77	62.0	1,521	74.3
Ischemic stroke	698	6.0	9	5.7	18	4.8	3	2.7	111	5.4
Peripheral artery disease	1734	14.9	20	12.6	58	15.5	19	15.3	325	15.9
Chronic kidney disease	524	4.5	8	5.0	15	4.0	5	4.0	90	4.4
COPD	1117	9.6	13	8.2	23	6.2	8	6.5	190	9.3
Heart failure	1653	14.2	20	12.6	37	9.9	11	8.9	276	13.5
Depression	1315	11.3	19	12.0	39	10.4	13	10.5	203	9.9
CCI score, mean (SD)	1.8 (1.4)		1.8 (1.6)		1.7 (1.4)		1.7 (1.4)		1.8 (1.6)	

*CCI* Charlson Comorbidity Index, *COPD* chronic obstructive pulmonary disease, *n* number, *SD* standard deviation

**Fig. 3:**
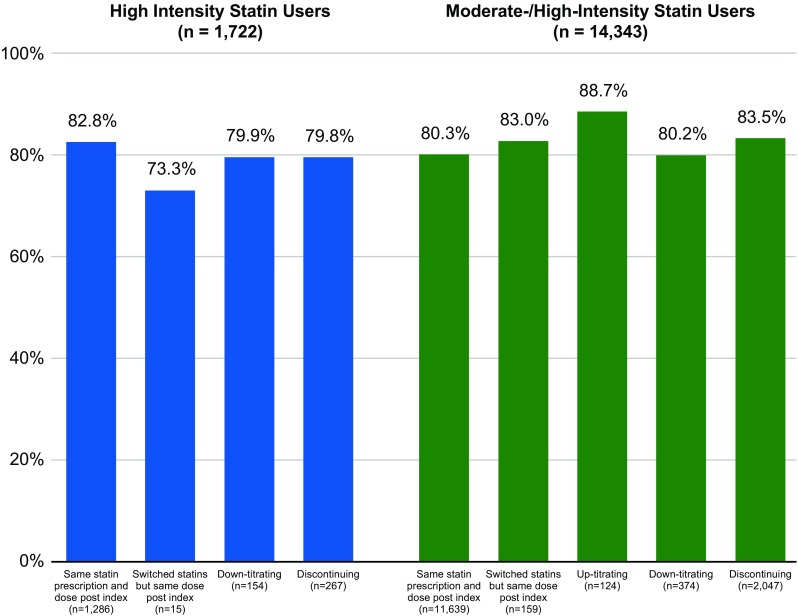
Proportion (%) of ASCVD patients using moderate-/high-intensity statins with LDL-C ≥ 70 mg/dL, by treatment patterns, 2014

Among all patients in the 2014 cohort who made no modifications to their index moderate- or high-intensity statin regimen, only 20% attained the LDL-C goal (Fig. 3, Supplementary Table 3). Among patients with other treatment changes, only 11–24% attained the LDL-C goal (Supplemental Table 3), with a similarly low percentage of patients in each of the treatment pattern groups attaining LDL-C < 70 mg/dL. LDL-C goal attainment results were also similar among patients using an index high-intensity statin. Only 17–19% of each annual cohort of patients with no changes to their high-intensity statin regimens achieved goal, and 11–26% of patients who modified their high-intensity statin regimens achieved the LDL-C goal.

## Discussion

This study demonstrates that a considerable proportion of ASCVD patients who are using moderate- or high-intensity statins are not reaching the LDL-C treatment goal of < 70 mg/dL and, therefore, remain at increased risk of cardiovascular events. The results of this study not only highlight suboptimal goal attainment among high-risk patients, but also add to a growing body of literature that has consistently documented low levels of LDL-C goal attainment in statin users more generally [15, 17, 18, 21–23]. It, therefore, seems likely that the observations from Germany reflect treatment patterns in other industrialized countries. Findings from the current study demonstrate that, for the majority of ASCVD patients who had filled prescriptions for moderate- or high-intensity statins, even the lowest documented LDL-C exceeded the ESC/EAS guideline-recommended level of < 70 mg/dL. Low rates of goal attainment occurred across the 3-year study period, and in all patient subgroups defined by key ASCVD comorbidities, including those with diabetes and multiple prior cardiovascular events. The rate of goal attainment remained low over time even with an increasing proportion of study patients using high-intensity statins.

Discontinuation of statin use was the commonly observed treatment modification, with 14–16% of all ASCVD patients discontinuing therapy during follow-up. While the rate of LDL-C goal attainment among patients who discontinued moderate- or high-intensity statin use was only 17% across all years, the rate of goal attainment was only marginally better (20%) among patients who remained on the same statin agent and dose. Although switches and up-titration of statin dose may represent attempts to improve patients’ ability to achieve the LDL-C goal, few patients exhibited those treatment modifications. The low occurrence of these treatment modifications may be indicative of clinicians’ desire to avoid prescribing high-intensity statins if possible, concerns about increased risk of diabetes [24, 25] and about statin tolerability at higher doses/intensity, or patients’ unwillingness to modify their therapy regimen [26].

The present results are not only consistent with those from previous observational studies, but provide more current data for a large sample of high-risk patients treated with moderate- or high-intensity statins. In the EUROASPIRE IV study, which included 6,648 patients with coronary heart disease in 24 European countries, only 19% achieved LDL-C goal, with goal attainment achieved by 14–16% of women and men on low-/moderate-intensity statins, and 20–29% of individuals on high-intensity statins [16]. Data collected in the 2008 Dyslipidemia International Survey (DYSIS) from 748 office-based physicians throughout Germany indicated that, despite use of statins generally in the range of simvastatin 20–40 mg daily or equivalent, 58% of all patients (*n* = 4282) and high-risk patients (*n* = 3783) did not attain LDL-C goals [27]. In another analysis of data collected in the cross-sectional, observational DYSIS study for 57,885 statin-treated outpatients in 30 countries across Europe, the Middle East, Africa, Asia, and Canada, patients were classified as being at very high, high, or non-high cardiovascular risk based on the 2011 ESC/EAS guidelines [7, 14]. Overall, only 27% of patients achieved their risk-based LDL-C goal. German patients exhibited the lowest level of goal attainment (14.3% of 3879 patients), with only 11% of very high-risk patients attaining LDL-C goal [28]. The impact of Germany’s statutory health insurance disease management programs (DMP) for diabetes mellitus and coronary heart disease on attainment of lipid-lowering goals has been assessed using data obtained in the prospective, non-interventional LIMA (Leitliniengerechte Lipidtherapie und Zielwerterreichung bei Risikopatienten im klinischen Alltag) study. In this secondary prevention population of 12,154 adults, 7–10% attained the < 70 mg/dL LDL-C goal at any point during a 12-month follow-up [29].

Similar goal attainment results were also reported for the retrospective cross-sectional DISCOVER study which assessed the rate of goal attainment in very high risk, ambulatory patients who were using generic atorvastatin (mean dose 27.9 ± 15.8 mg/day) [23]. Only 11% of patients in this population (patients with at least 1 month of stable treatment), diagnosed with diabetes mellitus (*n* = 658), coronary heart disease (*n* = 1233), or both (*n* = 734) reached LDL-C of < 70 mg/dL.

Statins are recommended as first-line therapy for patients with elevated LDL-C, and the majority of clinical guidelines, including the 2016 ESC/EAS Guidelines for the Management of Dyslipidaemias, recommend a ‘treat to target’ approach [6]. The low rate of LDL-C goal attainment observed in the present study population highlights an essential gap between the optimal, guideline-recommended LDL-C levels and actual levels achieved using moderate- or high-intensity statins. A number of factors may contribute to this observed lack of goal attainment. Not all patients with hyperlipidemia are able to tolerate statins or to achieve sufficient LDL-C reductions with statin therapy [30]. In particular, some patients with ASCVD or diabetes mellitus, and even some individuals who are high risk but asymptomatic, may be unable to reach LDL-C treatment goals even at the highest tolerated statin dose [6]. To ensure that patients are progressing toward LDL-C goals, the ESC/EAS guidelines recommend routine monitoring of statin compliance and treatment efficacy through laboratory assessment of LDL-C and other lipid measures, and physicians are urged to consider treatment modifications or augmentation if a patient’s therapeutic response is inadequate [6, 7]. Our results suggest that, in actual practice, treatment modifications rarely occurred in the ASCVD population, even in light of laboratory assessments indicating that the majority of patients using moderate-/high-intensity statins are not achieving LDL-C goals. Future research is needed to understand why treatment modifications are so rare, but the current findings highlight an important unmet need in the large number of patients who fail to achieve LDL-C goals, regardless of treatment pattern.

The consistently observed lack of goal attainment among statin users in Germany and elsewhere highlights the need for a range of therapeutic options to meet diverse patient needs. Proprotein convertase subtilisin/kexin type 9 (PCSK9) inhibitors, for example, represent a new therapeutic option that has recently entered the treatment landscape for patients with hyperlipidemia who are unable to achieve goal with statin monotherapy [31, 32].

The database used in this study is unique in providing access to detailed clinical data for over 2 million individuals across a multi-year period. From this large and representative population, we identified over 14,000 ASCVD patients in each year from 2012 to 2014. Patients contributed data in each study year for which they had an LDL-C value recorded in the database, which enabled us to examine patterns of LDL-C goal attainment in a largely stable population over time. The availability of follow-up data in this data source also allowed for the assessment of treatment patterns for the majority of study patients. As with any study, there are also limitations. Generalizability is an important consideration, and we note that our results may not generalize to the experience of all ASCVD patients since we focused exclusively on patients who were using either moderate- or high-intensity statins. We used the AHA and ACC classifications to define moderate- and high-intensity statins. Since this paper focuses primarily on results for the combined population of moderate-/high-intensity statin users, the distinction between these two levels of intensity is not critical. That said, definitions of statin intensity vary across geographies. If high intensity is defined more broadly (e.g., ≥ simvastatin 40 mg), a larger portion of each annual cohort would fall into the high-intensity category. German guidelines, for example, recommend simvastatin 40 mg as the standard dose for high-risk patients, which may explain why that majority of study patients were classified into the moderate-intensity category and may also have contributed to the low observed rate of up-titration.

In addition, the study design did not allow for evaluation of either the reasons for modifications of treatment regimens or why patients were unable to meet the LDL-C goal. Despite the very large sample size in this study, the number of high-intensity statin users included in the treatment patterns analysis was relatively small, so there may be greater variability in the estimates derived for that group.

The primary strength of the study is its use of a large nationwide database. Another strength is the use of real-world data on diagnoses in primary care practices; in this setting, diagnoses are continuously documented, allowing for unbiased assessment (i.e., no recall bias). Finally, the study design did not allow for consideration of the impact of treatment adherence on goal attainment. However, study patients had been prescribed moderate- or high-intensity statins and confirmed to have these statins on hand at the time that their lowest LDL-C values were obtained.

## Conclusions

LDL-C is an important and modifiable cardiovascular risk factor [1]. In routine clinical practice, however, our study suggests that approximately 80% of patients with ASCVD who use moderate- or high-intensity statins do not achieve the guideline-recommended LDL-C goal of < 70 mg/dL. In addition, few patients modified their statin regimens during follow-up and the majority of study patients were above LDL-C goal, regardless of the statin treatment pattern they exhibited. This lack of goal attainment, alongside a pattern of minimal treatment modifications, highlights an unmet clinical need as patients who remain above LDL-C goal remain at increased cardiovascular risk. With this low rate of LDL-C goal attainment among high-risk ASCVD patients treated with high- or moderate-intensity statins, alternative therapeutic options are needed to help patients achieve CV risk reduction.

## Electronic supplementary material

Below is the link to the electronic supplementary material.

Supplementary material 1 (DOCX 40 KB)

Supplementary material 2 (DOCX 40 KB)

Supplementary material 3 (DOCX 42 KB)

## References

[1] Collins R, Reith C, Emberson J, Armitage J, Baigent C, Blackwell L, Blumenthal R, Danesh J, Smith GD, DeMets D, Evans S, Law M, MacMahon S, Martin S, Neal B, Poulter N, Preiss D, Ridker P, Roberts I, Rodgers A, Sandercock P, Schulz K, Sever P, Simes J, Smeeth L, Wald N, Yusuf S, Peto R (2016). Interpretation of the evidence for the efficacy and safety of statin therapy. Lancet.

[2] Baigent C, Keech A, Kearney PM, Blackwell L, Buck G, Pollicino C, Kirby A, Sourjina T, Peto R, Collins R, Simes R, Cholesterol Treatment Trialists C (2005). Efficacy and safety of cholesterol-lowering treatment: prospective meta-analysis of data from 90,056 participants in 14 randomised trials of statins. Lancet.

[3] Cholesterol Treatment Trialists C, Baigent C, Blackwell L, Emberson J, Holland LE, Reith C, Bhala N, Peto R, Barnes EH, Keech A, Simes J, Collins R (2010). Efficacy and safety of more intensive lowering of LDL cholesterol: a meta-analysis of data from 170,000 participants in 26 randomised trials. Lancet.

[4] Cannon CP, Blazing MA, Giugliano RP, McCagg A, White JA, Theroux P, Darius H, Lewis BS, Ophuis TO, Jukema JW, De Ferrari GM, Ruzyllo W, De Lucca P, Im K, Bohula EA, Reist C, Wiviott SD, Tershakovec AM, Musliner TA, Braunwald E, Califf RM, Investigators I-I (2015). Ezetimibe added to statin therapy after acute coronary syndromes. N Engl J Med.

[5] Thavendiranathan P, Bagai A, Brookhart MA, Choudhry NK (2006). Primary prevention of cardiovascular diseases with statin therapy: a meta-analysis of randomized controlled trials. Arch Intern Med.

[6] Piepoli MF, Hoes AW, Agewall S, Albus C, Brotons C, Catapano AL, Cooney MT, Corra U, Cosyns B, Deaton C, Graham I, Hall MS, Hobbs FD, Lochen ML, Lollgen H, Marques-Vidal P, Perk J, Prescott E, Redon J, Richter DJ, Sattar N, Smulders Y, Tiberi M, van der BartWorp H, van Dis I, Verschuren WM (2016). 2016 European Guidelines on cardiovascular disease prevention in clinical practice: the Sixth Joint Task Force of the European Society of Cardiology and Other Societies on Cardiovascular Disease Prevention in Clinical Practice (constituted by representatives of 10 societies and by invited experts) Developed with the special contribution of the European Association for Cardiovascular Prevention & Rehabilitation (EACPR). Atherosclerosis.

[7] Reiner Z, Catapano AL, De Backer G, Graham I, Taskinen MR, Wiklund O, Agewall S, Alegria E, Chapman MJ, Durrington P, Erdine S, Halcox J, Hobbs R, Kjekshus J, Filardi PP, Riccardi G, Storey RF, Wood D, European Association for Cardiovascular P, Rehabilitation, Guidelines ESCCfP, Committees (2011). ESC/EAS Guidelines for the management of dyslipidaemias: the task force for the management of dyslipidaemias of the European Society of Cardiology (ESC) and the European Atherosclerosis Society (EAS). Eur Heart J.

[8] Catapano AL, Graham I, De Backer G, Wiklund O, Chapman MJ, Drexel H, Hoes AW, Jennings CS, Landmesser U, Pedersen TR, Reiner Z, Riccardi G, Taskinen MR, Tokgozoglu L, Verschuren WM, Vlachopoulos C, Wood DA, Zamorano JL, Authors/Task Force M, Additional C (2016). 2016 ESC/EAS guidelines for the management of dyslipidaemias. Eur Heart J.

[9] De Vera MA, Bhole V, Burns LC, Lacaille D (2014). Impact of statin adherence on cardiovascular disease and mortality outcomes: a systematic review. Br J Clin Pharmacol.

[10] Bitton A, Choudhry NK, Matlin OS, Swanton K, Shrank WH (2013). The impact of medication adherence on coronary artery disease costs and outcomes: a systematic review. Am J Med.

[11] Colantonio LD, Huang L, Monda KL, Bittner V, Serban MC, Taylor B, Brown TM, Glasser SP, Muntner P, Rosenson RS (2017). Adherence to high-intensity statins following a myocardial infarction hospitalization among medicare beneficiaries. JAMA Cardiol.

[12] Degli Esposti L, Saragoni S, Batacchi P, Benemei S, Geppetti P, Sturani A, Buda S, Degli Esposti E (2012). Adherence to statin treatment and health outcomes in an Italian cohort of newly treated patients: results from an administrative database analysis. Clin Ther.

[13] Fox KM, Gandhi SK, Ohsfeldt RL, Davidson MH (2007). Comparison of low-density lipoprotein cholesterol reduction after switching patients on other statins to rosuvastatin or simvastatin in a real-world clinical practice setting. Am J Manag Care.

[14] Gitt AK, Lautsch D, Ferrieres J, Kastelein J, Drexel H, Horack M, Brudi P, Vanneste B, Bramlage P, Chazelle F, Sazonov V, Ambegaonkar B (2016). Low-density lipoprotein cholesterol in a global cohort of 57,885 statin-treated patients. Atherosclerosis.

[15] Chiang CE, Ferrieres J, Gotcheva NN, Raal FJ, Shehab A, Sung J, Henriksson KM, Hermans MP (2016). Suboptimal control of lipid levels: results from 29 countries participating in the centralized pan-regional Surveys on the undertreatment of hypercholesterolaemia (CEPHEUS). J Atheroscler Thromb.

[16] Reiner Z, De Backer G, Fras Z, Kotseva K, Tokgozoglu L, Wood D, De Bacquer D, Investigators E (2016). Lipid lowering drug therapy in patients with coronary heart disease from 24 European countries–Findings from the EUROASPIRE IV survey. Atherosclerosis.

[17] Kotseva K, Wood D, De Backer G, De Bacquer D, Pyorala K, Keil U, Group ES (2009). Cardiovascular prevention guidelines in daily practice: a comparison of EUROASPIRE I, II, and III surveys in eight European countries. Lancet.

[18] Kotseva K, Wood D, De Bacquer D, De Backer G, Ryden L, Jennings C, Gyberg V, Amouyel P, Bruthans J, Castro Conde A, Cifkova R, Deckers JW, De Sutter J, Dilic M, Dolzhenko M, Erglis A, Fras Z, Gaita D, Gotcheva N, Goudevenos J, Heuschmann P, Laucevicius A, Lehto S, Lovic D, Milicic D, Moore D, Nicolaides E, Oganov R, Pajak A, Pogosova N, Reiner Z, Stagmo M, Stork S, Tokgozoglu L, Vulic D, Investigators E (2016). EUROASPIRE IV: a European Society of Cardiology survey on the lifestyle, risk factor and therapeutic management of coronary patients from 24 European countries. Eur J Prev Cardiol.

[19] Quek RG, Fox KM, Wang L, Li L, Gandra SR, Wong ND (2016). A US claims-based analysis of real-world lipid-lowering treatment patterns in patients with high cardiovascular disease risk or a previous coronary event. Am J Cardiol.

[20] Charlson M, Szatrowski TP, Peterson J, Gold J (1994). Validation of a combined comorbidity index. J Clin Epidemiol.

[21] Jones PH, Nair R, Thakker KM (2012). Prevalence of dyslipidemia and lipid goal attainment in statin-treated subjects from 3 data sources: a retrospective analysis. J Am Heart Assoc.

[22] Karlson BW, Nicholls SJ, Lundman P, Palmer MK, Barter PJ (2013). Achievement of 2011 European low-density lipoprotein cholesterol (LDL-C) goals of either < 70 mg/dl or ≥ 50% reduction in high-risk patients: results from VOYAGER. Atherosclerosis.

[23] Laufs U, Karmann B, Pittrow D (2016). Atorvastatin treatment and LDL cholesterol target attainment in patients at very high cardiovascular risk. Clin Res Cardiol.

[24] Mansi IA, Frei CR, Halm EA, Mortensen EM (2017). Association of statins with diabetes mellitus and diabetic complications: role of confounders during follow-up. J Investig Med.

[25] Preiss D, Seshasai SR, Welsh P, Murphy SA, Ho JE, Waters DD, DeMicco DA, Barter P, Cannon CP, Sabatine MS, Braunwald E, Kastelein JJ, de Lemos JA, Blazing MA, Pedersen TR, Tikkanen MJ, Sattar N, Ray KK (2011). Risk of incident diabetes with intensive-dose compared with moderate-dose statin therapy: a meta-analysis. JAMA.

[26] Spence JD, Dresser GK (2016) Overcoming Challenges With Statin Therapy. J Am Heart Assoc 5 (1). 10.1161/JAHA.115.002497 10.1161/JAHA.115.002497 PMC4859367 26819251

[27] Gitt AK, Junger C, Smolka W, Bestehorn K (2010). Prevalence and overlap of different lipid abnormalities in statin-treated patients at high cardiovascular risk in clinical practice in Germany. Clin Res Cardiol.

[28] Gitt AK, Lautsch D, Ferrieres J, Kastelein J, Drexel H, Horack M, Brudi P, Vanneste B, Bramlage P, Chazelle F, Sazonov V, Ambegaonkar B (2016). Contemporary data on low-density lipoprotein cholesterol target value attainment and distance to target in a cohort of 57,885 statin-treated patients by country and region across the world. Data Brief.

[29] Gitt AK, Sonntag F, Jannowitz C, Weizel A, Karmann B, Schaefer JR, Pittrow D, Hildemann SK (2016). Better lipid target achievement for secondary prevention through disease management programs for diabetes mellitus and coronary heart disease in clinical practice in Germany. Curr Med Res Opin.

[30] Drakopoulou M, Toutouzas K, Stathogiannis K, Synetos A, Trantalis G, Tousoulis D (2016). Managing the lipid profile of coronary heart disease patients. Expert Rev Cardiovasc Ther.

[31] Sabatine MS (2017). Proprotein convertase subtilisin/kexin type 9 (PCSK9) inhibitors: comparing and contrasting guidance across the Atlantic. Eur Heart J.

[32] Sabatine MS, Giugliano RP, Keech AC, Honarpour N, Wiviott SD, Murphy SA, Kuder JF, Wang H, Liu T, Wasserman SM, Sever PS, Pedersen TR, Committee FS, Investigators (2017). Evolocumab and clinical outcomes in patients with cardiovascular disease. N Engl J Med.

