# Supplementary material for: Treatment patterns and low-density lipoprotein cholesterol (LDL-C) goal attainment among patients receiving high- or moderate-intensity statins
Source: Clin Res Cardiol. 2017 Dec 22;107(5):380–8. doi: 10.1007/s00392-017-1193-z (PMC5913378; doi:10.1007/s00392-017-1193-z)
Supplement: Supplementary file 1 — Supplementary material 1 (DOCX 40 KB) [file 392_2017_1193_MOESM1_ESM.docx]

Treatment patterns and low-density lipoprotein cholesterol (LDL-C) goal attainment among patients receiving high- or moderate-intensity statins

Kathleen M. Fox, PhD^1^, Ming-Hui Tai, PhD^2^, Karel Kostev, MsD, PhD^3^, Maximilian Hatz, PhD^2^, Yi Qian, PhD^2^, Ulrich Laufs, MD^4^

Affiliations: ^1^Strategic Healthcare Solutions, LLC, Aiken, SC, USA; ^2^Amgen, Inc, Thousand Oaks, CA, USA; ^3^QuintilesIMS Frankfurt, Germany; ^4^Klinik und Poliklinik für Kardiologie, Universitätsklinikum Leipzig, Leipzig, Germany

Correspondence to: Ming-Hui Tai, MS, PhD

Email: mtai@amgen.com

**Supplementary Table1. Statin Treatment Intensity Definitions**

| **Statin Treatment** | **Average Daily Dose*** | | |
| --- | --- | --- | --- |
|  | **Low-intensity** | **Moderate-intensity** | **High-intensity** |
| **Atorvastatin** | n/a | <30 | ≥30 |
| **Fluvastatin** | <60 | ≥60 | n/a |
| **Lovastatin** | <30 | ≥30 | n/a |
| **Pitavastatin** | <1.5 | ≥1.5 | n/a |
| **Pravastatin** | <30 | ≥30 | n/a |
| **Rosuvastatin** | n/a | <15 | ≥15 |
| **Simvastatin** | <15 | 15-60 | ≥60 |

Adapted from the 2013 American Heart Association and American College of Cardiology guidelines on the treatment of blood cholesterol to reduce atherosclerotic cardiovascular risk in adults.

*Average Daily Dose = (Strength of Statin * Quantity) / Days Supply
