# Supplementary material for: Treatment patterns and low-density lipoprotein cholesterol (LDL-C) goal attainment among patients receiving high- or moderate-intensity statins
Source: Clin Res Cardiol. 2017 Dec 22;107(5):380–8. doi: 10.1007/s00392-017-1193-z (PMC5913378; doi:10.1007/s00392-017-1193-z)
Supplement: Supplementary file 2 — Supplementary material 2 (DOCX 40 KB) [file 392_2017_1193_MOESM2_ESM.docx]

Treatment patterns and low-density lipoprotein cholesterol (LDL-C) goal attainment among patients receiving high- or moderate-intensity statins

Kathleen M. Fox, PhD^1^, Ming-Hui Tai, PhD^2^, Karel Kostev, MsD, PhD^3^, Maximilian Hatz, PhD^2^, Yi Qian, PhD^2^, Ulrich Laufs, MD^4^

Affiliations: ^1^Strategic Healthcare Solutions, LLC, Aiken, SC, USA; ^2^Amgen, Inc, Thousand Oaks, CA, USA; ^3^QuintilesIMS Frankfurt, Germany; ^4^Klinik und Poliklinik für Kardiologie, Universitätsklinikum Leipzig, Leipzig, Germany

Correspondence to: Ming-Hui Tai, MS, PhD

Email: mtai@amgen.com

**Supplementary Table 2. Number of Patients and Proportion (%) of ASCVD Patients with LDL-C ≥70 mg/dL, Full Study Population and Subgroups by Baseline Clinical Characteristics**

| **Population** | **2012** | | **2013** | | **2014** | |
| --- | --- | --- | --- | --- | --- | --- |
|  | **Total N** | **%** | **Total N** | **%** | **Total N** | **%** |
| Patients total | 14,058 | 80.3 | 15,383 | 80.4 | 16,316 | 80.5 |
| Myocardial infarction in past year | 3,232 | 79.3 | 3,608 | 78.5 | 3,664 | 78.7 |
| Ischemic stroke in past year | 736 | 73.8 | 834 | 77.7 | 952 | 77.5 |
| Peripheral artery disease | 1,636 | 79.1 | 1,725 | 79.5 | 1,919 | 81.0 |
| Chronic kidney disease | 549 | 73.4 | 641 | 76.1 | 760 | 75.3 |
| Type 2 diabetes | 4,409 | 74.1 | 4,650 | 75.0 | 5,047 | 74.5 |
| ≥ 2 prior CV events | 401 | 75.1 | 423 | 71.9 | 434 | 75.6 |
| Diabetes and ≥ 2 prior CV events | 174 | 72.4 | 185 | 67.0 | 200 | 66.5 |
| Ezetimibe use | 1,336 | 73.8 | 1209 | 74.9 | 1,107 | 77.1 |

ASCVD: atherosclerotic cardiovascular disease; CV: cardiovascular
