# Supplementary material for: Treatment patterns and low-density lipoprotein cholesterol (LDL-C) goal attainment among patients receiving high- or moderate-intensity statins
Source: Clin Res Cardiol. 2017 Dec 22;107(5):380–8. doi: 10.1007/s00392-017-1193-z (PMC5913378; doi:10.1007/s00392-017-1193-z)
Supplement: Supplementary file 3 — Supplementary material 3 (DOCX 42 KB) [file 392_2017_1193_MOESM3_ESM.docx]

Treatment patterns and low-density lipoprotein cholesterol (LDL-C) goal attainment among patients receiving high- or moderate-intensity statins

Kathleen M. Fox, PhD^1^, Ming-Hui Tai, PhD^2^, Karel Kostev, MsD, PhD^3^, Maximilian Hatz, PhD^2^, Yi Qian, PhD^2^, Ulrich Laufs, MD^4^

Affiliations: ^1^Strategic Healthcare Solutions, LLC, Aiken, SC, USA; ^2^Amgen, Inc, Thousand Oaks, CA, USA; ^3^QuintilesIMS Frankfurt, Germany; ^4^Klinik und Poliklinik für Kardiologie, Universitätsklinikum Leipzig, Leipzig, Germany

Correspondence to: Ming-Hui Tai, MS, PhD

Email: mtai@amgen.com

**Supplementary Table 3. Number of Patients and Proportion (%) of ASCVD Patients with LDL-C ≥70 mg/dL, by Treatment Patterns and Annual Cohort**

|  | 2012 | | 2013 | | 2014 | |
| --- | --- | --- | --- | --- | --- | --- |
| **Treatment Patterns** | Total N | % | Total N | % | Total N | % |
| **High-intensity statin** |  |  |  |  |  |  |
| Patients total | 1,045 | 79.5 | 1,516 | 81.0 | 1,722 | 82.0 |
| Same statin prescription and dose post index | 669 | 81.0 | 1,045 | 81.4 | 1,286 | 82.8 |
| Other statin prescription but same dose post index | 24 | 87.5 | 18 | 77.8 | 15 | 73.3 |
| Down-titrating | 97 | 78.4 | 147 | 81.6 | 154 | 79.9 |
| Discontinuing | 255 | 75.3 | 306 | 79.4 | 267 | 79.8 |
|  |  |  |  |  |  |  |
| **High- or moderate-intensity statin** |  |  |  |  |  |  |
| Patients total | 13,017 | 80.6 | 14,180 | 80.9 | 14,343 | 80.9 |
| Same statin prescription and dose post index | 10,231 | 80.0 | 11,360 | 80.4 | 11,639 | 80.3 |
| Other statin prescription but same dose post index | 187 | 75.9 | 189 | 84.1 | 159 | 83.0 |
| Up-titrating | 130 | 88.5 | 128 | 85.9 | 124 | 88.7 |
| Down-titrating | 385 | 82.6 | 403 | 80.6 | 374 | 80.2 |
| Discontinuing | 2,084 | 83.0 | 2,100 | 83.1 | 2,047 | 83.5 |

ASCVD: atherosclerotic cardiovascular disease
